# Efficient production of a cyclic dipeptide (*cyclo*-TA) using heterologous expression system of filamentous fungus *Aspergillus oryzae*

**DOI:** 10.1186/s12934-022-01872-8

**Published:** 2022-07-18

**Authors:** Jianzhao Qi, Haiyan Han, Dan Sui, Shengnan Tan, Changli Liu, Pengchao Wang, Chunliang Xie, Xuekui Xia, Jin-ming Gao, Chengwei Liu

**Affiliations:** 1 Key Laboratory for Enzyme and Enzyme-Like Material Engineering of Heilongjiang, College of Life Science, Northeast Forestry University, HarbinHeilongjiang, 150040 China; 2 Shaanxi Key Laboratory of Natural Products & Chemical Biology, College of Chemistry & Pharmacy, Northwest A&F University, Yangling, 712100 Shaanxi China; 3 Institute of Bast Fiber Crops, Chinese Academy of Agricultural Sciences, Changsha, 410205 Hunan China; 4 Biology Institute, Qilu University of Technology (Shandong Academy of Sciences), Jinan, 250103 Shandong China

**Keywords:** *Aspergillus oryzae*, Cyclic dipeptide, Echinulin, Heterologous expression, NRPS

## Abstract

**Background:**

Cyclic dipeptides are an important class of natural products owing to their structural diversity and biological activities. In fungi, the cyclo-ring system is formed through the condensation of two α-amino acids via non-ribosomal peptide synthetase (NRPS). However, there are few investigations on the functional identification of this enzyme. Additionally, information on how to increase the production of cyclic dipeptide molecules is relatively scarce.

**Results:**

We isolated the *Eurotium cristatum* NWAFU-1 fungus from Jing-Wei Fu brick tea, whose fermentation metabolites contain echinulin-related cyclic dipeptide molecules. We cloned the *cirC* gene, encoding an NRPS, from *E. Cristatum* NWAFU-1 and transferred it into the heterologous host *Aspergillus oryzae*. This transformant produced a novel metabolite possessing an l-tryptophan-l-alanine cyclic dipeptide backbone (*Cyclo*-TA). Based on the results of heterologous expression and microsomal catalysis, CriC is the first NRPS characterized in fungi that catalyzes the formation of a cyclic dipeptide from l-tryptophan and l-alanine. After substrate feeding, the final yield reached 34 mg/L. In this study, we have characterized a novel NRPS and developed a new method for cyclic dipeptide production.

**Conclusions:**

In this study we successfully expressed the *E. Cristatum* NWAFU-1 *criC* gene in *A. oryzae* to efficiently produce cyclic dipeptide compounds. Our findings indicate that the *A. oryzae* heterologous expression system constitutes an efficient method for the biosynthesis of fungal Cyclic dipeptides.

**Supplementary Information:**

The online version contains supplementary material available at 10.1186/s12934-022-01872-8.

## Background

Cyclic dipeptides ring systems are achieved by the fusion of two α-amino acids, and the fungi are well-known primary producers of a diversity of cyclic dipeptides [1–4]. They have a wide range of biological activities, including antimicrobial, antiviral, anticancer, and proangiogenic activity [4–6]. This type of natural product contains numerous therapeutically promising compounds [7], such as plinabulin, the derivative of phenylahistin which was isolated from the fungus *Aspergillus ustus* [8], plinabulin has been advanced to Phase III clinical trials as an antitumor drug candidate [9]. Even though medicinal chemists have widely developed and synthesized cyclic dipeptides, the understanding and manipulating their biosynthetic pathways results in the formation of new chemical structures, that may lead to the production of new active compounds [10].

**Fig. 1:**
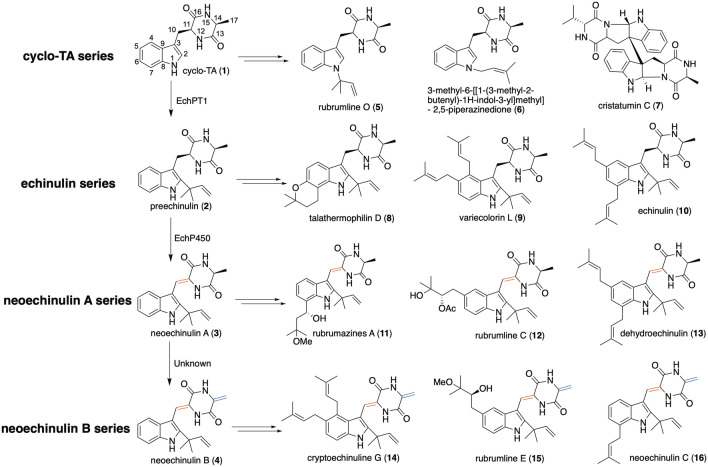
Representative natural products containing the Cyclo-TA scaffold

The *l*-tryptophan-*l*-alanine cyclic dipeptide (*Cyclo*-TA (**1**) series) is a structural moiety containing an indole diketopiperazine (DKP) scaffold. A reverse C2 prenylation (preechinulin (**2**), echinulin series) and unsaturated derivatives between C10 and C11 (*Δ*^10^) (neoechinulin A (**3**) series) and C14 and C17 (*Δ*^14^) (neoechinulin B (**4**) series) constitute the main structural modifications to the *Cyclo*-TA backbone [11]. Prenylation of the backbone by prenyltransferase is the most frequent modification occurring in tryptophan-containing cyclic dipeptides, and the varying degrees (one to four prenyl moieties) and positions (C-2/4/5/6/7 or N-1) of phenyl modifications that appear on the backbone expand the structural diversity [12, 13]. Reverse and regular prenylated compounds, such as rubrumline O (**5**) and 3-methyl-6-[[1-(3-methyl-2-butenyl)-1H-indol-3-yl]methyl]-2,5-piperazinedione (**6**) with an N-1 modification of the *Cyclo*-TA backbone are inhibitors of influenza viruses [14]. *Cyclo*-TA that has not undergone prenylation can be closed with a C-N bond between C-2 and N-12 to form a 6-5-5-6 ring system and further form a heterodimer, such as cristatumin C (**7**) [15]. Talathermophilin D (**8**) represents a class of pyranoindole modifications at the C-6 and C-7 positions, which are uncommon in natural DKP products [16]. Variecolorin L (**9**) [17] and echinulin (**10**) both contain two dimethylallyl (DMA) moieties at the C-4/5 and C-5/7 positions, respectively, as well as an isopentenyl moiety at C-2. Hydroxylation and oxy-methylation (e.g., rubrumazines A (**11**)) [18] or acetylation (e.g., rubrumline C (**12**) [14]) of DMA moieties and the presence of one (dehydroechinulin (**13**)) [19] or two (cryptoechinuline G (**14**) [20], rubrumline E (**15**), and neoechinulin C (**16**)) [11, 14] backbone double bonds enrich the diversity of compounds. Furthermore, post-modifications occurring on the prenyl moieties increase the structural diversity of *Cyclo-*TA containing compounds (Fig. 1). These compounds have been mainly isolated from different *Penicillium* [21, 22], *Aspergillus* [20, 23], and *Eurotium* [14, 18, 19] species.

Recently, Nies *et al.* successfully identified a biosynthetic gene cluster (BGC) for echinulin in *A. ruber* [11]. This cluster comprises four genes that are responsible for the backbone's components, including the coding region for a non-ribosomal peptide synthetase (NRPS, echPS), a cytochrome P450 enzyme (echP450), and two prenyltransferases (echPT1 and echPT2). In an in vitro enzyme activity experiment, EchPT1 was responsible for indole ring C2 reverse-prenylation, EchP450 for forming a double bond, and EchPT2 for the multi-prenylation at the C-5, 6, or 7 positions [11, 12]. Although NRPS is speculated to be the responsible enzyme for cyclic dipeptide production, its function has not been independently analyzed and verified.

In this study, we isolated a fungus from Jing-Wei Fu brick tea, which was identified by internal transcribed spacer (ITS) sequence analysis and named *Eurotium cristatum* NWAFU-1. The ability to generate cyclic dipeptide-related molecules was discovered upon analysis of the metabolites from fungal fermentation. Through analysis of the genomic data, we identified a gene cluster containing NRPS, which was predicted to be involved in the synthesis of *Cyclo*-TA. Furthermore, we describe the production of a cyclic dipeptide from transformants containing the core NRPS gene (*criC*) in the heterologous expression host *A. oryzae*.

## Results and discussion

### Identification of the *Cri* gene cluster for echinulin biosynthesis in *E. cristatum*

Jing-Wei Fu brick tea is a unique post-fermented tea product that is naturally co-fermented by microorganisms and has gained global popularity due to its potential health benefits. It naturally produces golden particles, commonly referred to as “golden flowers”, and contains a symbiotic fungus named *E. cristatum* [24]. We isolated the fungus *E. cristatum* NWAFU-1 from a brick tea sample collected from Xianyang City, Shaanxi Province, China (Additional file 1: Fig. S1). ITS sequence analysis of the ribosomal DNA was highly similar (99.43%) to the GenBank sequences from the fungus *E. cristatum* YKY807.

First, we examined the metabolites of *E. cristatum* NWAFU-1 by ultraperformance liquid chromatography–electrospray ionization–high resolution mass spectrometry (UPLC–ESI–HRMS) via classical or feature-based molecular networking workflows with the Global Natural Products Social Molecular Networking (GNPS, http://gnps.ucsd.edu) web platform. The molecular masses of 326.19, 394.25, 462.31, and 490.34 were detected, suggesting that these compounds could be echinulin and its derivatives (Additional file 1: Fig. S2).

To determine the BGC responsible for the production of cyclic dipeptide related compounds, we employed an independent study for genome mining on *E. cristatum* YKY807, using gene cluster search methods (a local BLAST search and the 2ndFind program [25]). We identified a putative BGC (*cri*), localized on a continuous DNA region of 27.4 kb that encoded seven putative enzymes: one NRPS (CriC), two annotated prenyltransferases (CriA and CriF), one cytochrome P450 (CriE), one FMN oxidoreductase (CriG), one transporter (CriB), and one functionally unknown protein (Additional file 1: Fig. S3).

Sequence similarity network analysis showed that CriC is grouped with NRPSs, suggesting a common role in the biosynthesis of cyclic dipeptides. Orthologous CriC proteins are involved in the biosynthesis of the structurally related cyclic dipeptide (AtaP/GliP/SirP/AclP/VerP) [26–30]. These observations indicate that sequence similarity network analysis can be used to predict the function of CriC family proteins with close similarity (Additional file 1: Fig. S4).

### Functional analysis of CriC

**Fig. 2:**
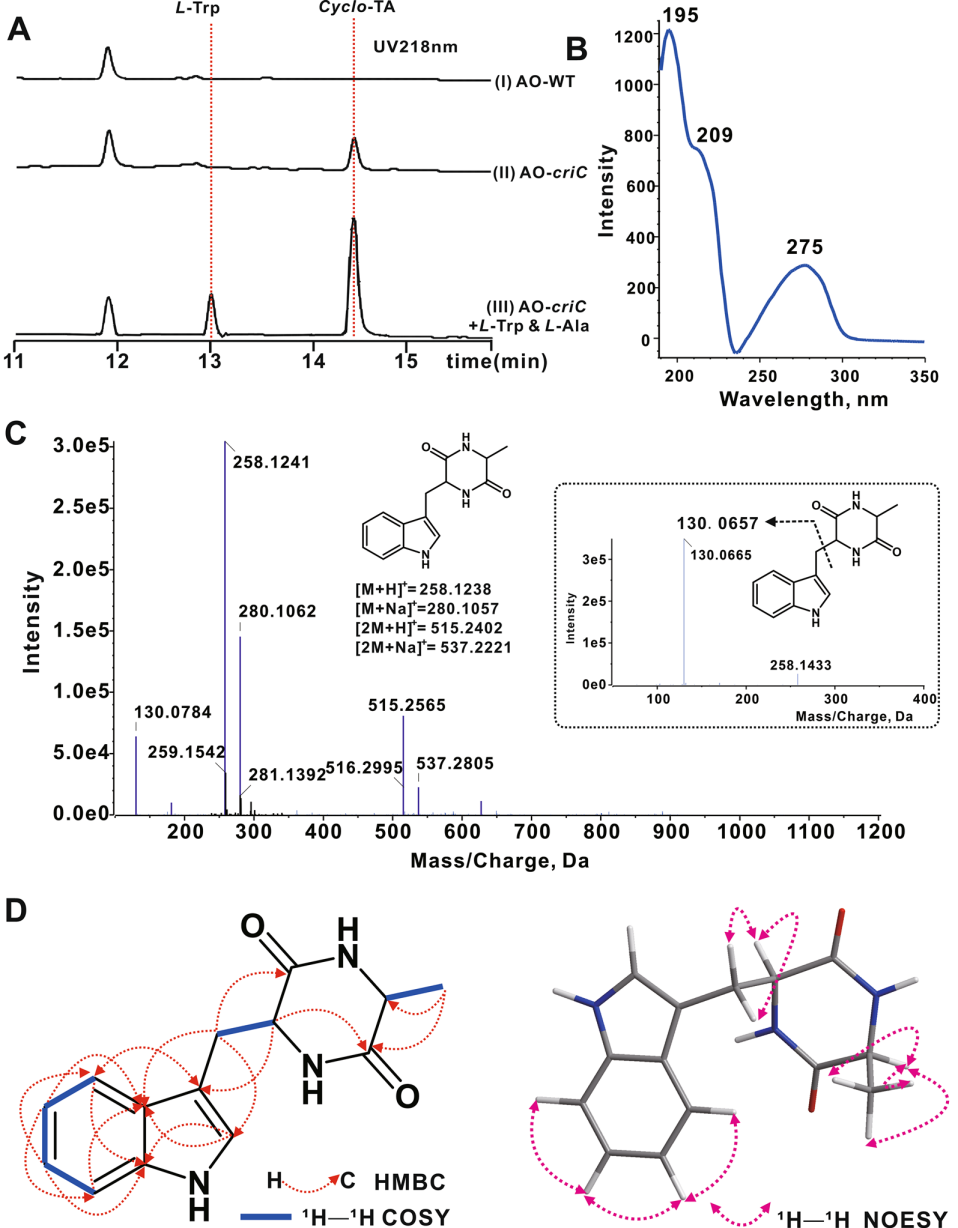
Heterologous expression of *criC* and characterization of metabolites. **A** HPLC trace of criC heterologous expression in *A. oryzae* NSAR1. (I) AO-WT (*A. oryzae* wild type), (II) metabolites produced by transformant AO-*criC*, (III) AO-*criC* feed by substrates (*l*-Trp and *l*-Ala). **B** UV absorption spectroscopy of criC product. **C** MS and MS/MS spectrum of CriC product. **D** Summary of HMBC, COSY, and NOESY experiments of the AO-*criC* product.

To examine the function of NRPS, the *criC* gene was amplified from *E. cristatum* NWAFU-1 genomic DNA, the purified PCR fragment was cloned into a pUSA2 plasmid [31], and transformed into *A. oryzae* NSAR1 to form AO-*criC* transformants. HPLC analysis of the partially purified fraction of AO-*criC* mycelial extracts showed a new peak, which was not found in the control culture of the wild-type strain (Fig. 2A). To determine the structure of this novel compound, the mycelial ethyl acetate extract of AO-*criC* was obtained by large-scale incubation in MPY medium. The crude extract was purified with silica gel column chromatography and partitioned with hexane–ethyl acetate. HPLC analysis resulted in the pure product *Cyclo*-TA (**1**) at a concentration of 7.5 mg/L. The obtained UV/Vis spectrum (maxima at λ = 195, 209, and 275 nm) was in agreement with the absorption characteristics of the indole ring (Fig. 2B). The molecular formula was determined to be C_14_H_16_N_3_O_2_ (calcd: 258.1238 [M + H]^+^, 280.1057 [M + Na]^+^ and found: 258.1241, 280.1062) by high resolution–electrospray ionization–mass spectrometry (HR-ESI–MS) analysis. A molecular weight of [2M + H]^+^ and [2M + Na]^+^ was also detected. MS/MS data showed a specific fragment of 130.0657 (an indole core ion) that was identified as a fragment of *Cyclo*-TA (Fig. 2C).

^13^C nuclear magnetic resonance (NMR) indicated two ketones (*δc* 169.5 & *δc* 170.6) carbonyl groups, suggesting that the structure of the compound is closely related to that of cyclic dipeptides. Extensive NMR data analysis, including HSQC, HMBC, COSY, and NOESY, confirmed the structure of **1**, as shown in Fig. 2D (Additional file 1: Figs. S5–10, Table S1).

### Biochemical characterization of CriC and improving *Cyclo*-TA production

**Fig. 3:**
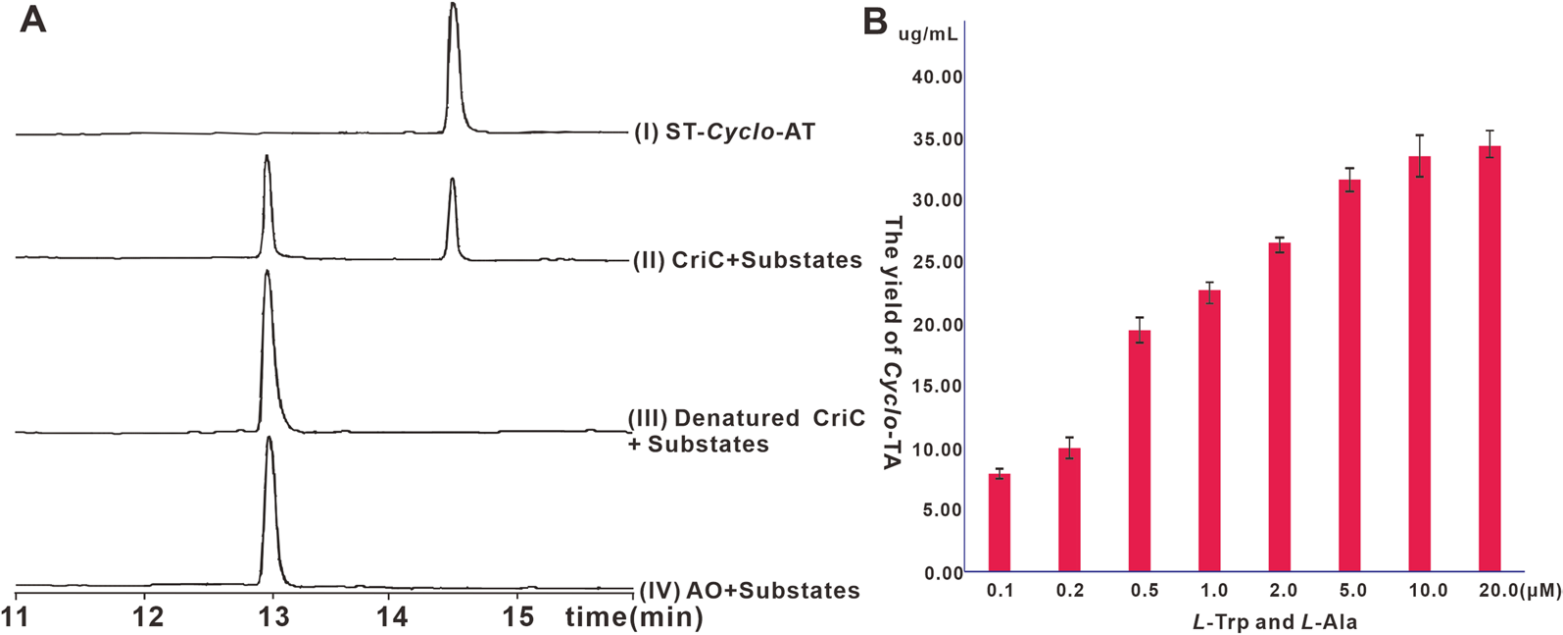
Biochemical characterization and improved production capacity of CriC. **A** Biochemical characterization of CriC microsome, (I) Standard *cyclo*-TA, (II) reaction mix containing CriC microsome and substrates (*l*-Trp and *l*-Ala), (III) reaction mix containing denatured CriC microsome and substrates, (iv) reaction mix containing substrates and microsome prepared from AO host. **B** Substrate feeding increases the yield of AO-*criC*. The substrate *l*-Trp and *l*-Ala was added in equal amounts for each gradient

The substrate specificity of CriC was also investigated. We performed an in vitro analysis using microsomes of AO-*criC* strains. Along with ATP, l-Trp and l-Ala were used as substrates in the reaction, which confirmed CriC's substrate specificity for these two amino acids (Fig. 3A). Using a time-dependent in vitro assay with both l-Trp and l-Ala as substrates, the final product increased over time (Additional file 1: Fig. S11). Using l-Trp or l-Ala as a fixed substrate to react with 19 other native amino acids from microsomes yielded no product (Additional file 1: Fig. S12).

Next, we investigated the potential of *A. oryzae* expressing recombinant *criC* for the industrial production of *Cyclo*-TA. As AO-*criC* transformants can efficiently produce the target product, we investigated the effects of increased yields of feed substrates (*l*-Trp and *l*-Ala) during culture. When different concentrations of the two substrates were added to the fermentation medium, the amount of the final product was significantly increased; when 20 μM of the two substrates were added separately, the yield reached 34 mg/L (Fig. 3B, Additional file 1: Fig. S13).

### Phylogenetic analysis of cyclic dipeptide synthetase

Based on the PKS/NRPS analysis website, CriC encodes an NRPS protein of 2127 amino acids that consist of two sets of domains for adenylation (A), thiolation (T), and condensation (C) (Additional file 1: Fig. S14A, B). The **A** domain chooses and activates a carboxylic acid substrate through adenylation following consumption of ATP, and then forms a thioester linkage by transferring the acyl group to the phosphopantetheinyl arm linked to the **T** domain. The **C** domain is involved in the formation of peptide bonds between two adjacent modules [32]. Correlation analysis for the adenylation domain of CriC and TaqA, an enzyme that binds anthranilate and two amino acids (l-Trp and l-Ala), showed that they act as substrates in the synthesis of fumiquinazoline F [33]. A comparison of the two proteins revealed that the domains of binding amino acids were predicted to have similar functions. The proposed biosynthetic reaction mechanism is shown in Additional file 1: Figure S14C.

**Fig. 4:**
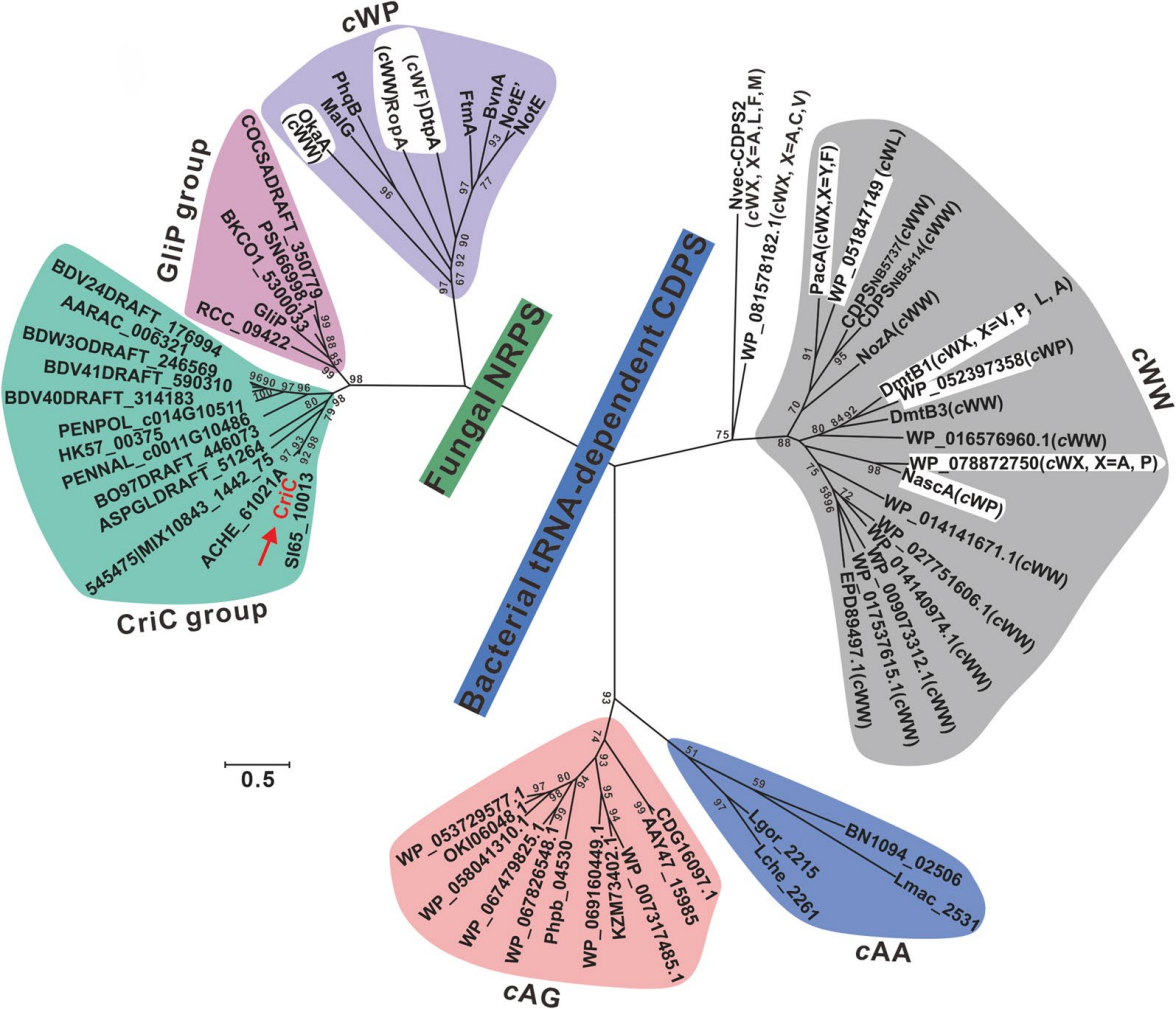
Phylogenetic analysis of CriC. Phylogenetic analysis of CriC and Cyclic dipeptide synthase with tryptophan or alanine as a substrate. *c*WW, *c*WL, *c*WA, *c*WP, *c*WV refer to cyclic dipeptides formed by tryptophan with tryptophan, leucine, alanine, proline, and valine, respectively, while *c*AA and *c*AG refer to cyclic dipeptides formed by alanine with alanine and glycine, respectively. Phylogenetic trees were constructed using MEGA X the maximum likelihood method

We constructed a phylogenetic tree using cyclic dipeptide proteins from BLAST analysis. The cyclic peptides ring system is generated by the condensation of two amino acids via two different pathways: via NRPS, which uses and activates free amino acids through adenylation [34, 35], or via cyclic dipeptide synthases (CDPs). CDPs kidnap aminoacyl-transfer RNAs (aa-tRNAs) from their primary use in the translation process [36]. According to phylogenetic tree analysis, enzymes that form cyclic dipeptides using tryptophan and/or alanine as substrates can be divided into two groups: bacterial-derived aa-tRNA-dependent cyclic dipeptide synthases and fungal-derived NRPSs. CriC differs from other reported NRPSs using tryptophan or alanine as substrates in that it forms a new branch with 14 other sequences from the National Center for Biotechnology Information (NCBI) whose function has not been reported (Fig. 4, Additional file 1: Table S2). Based on genome mining of a BGC for echinulin biosynthesis, we identified 15 fungal strains, depending on the presence of BGCs for non-ribosomal peptide biosynthesis, and a possible cluster for cyclic dipeptide biosynthesis. Additional file 1: Figure S15 shows 15 other gene clusters from *Eurotium*, *Aspergillus*, and *Penicillium* species.

## Conclusions

In summary, we showed that the *criC* gene, located in the echinulin BGC in *E. cristatum*, encodes an NRPS that catalyzes the condensation of *l*-Trp and *l*-Ala to produce *Cyclo*-TA (**1**) [37]. Phylogenetic trees show that it belongs to a distinct family, and similar gene clusters are found in *Eurotium*, *Penicillium*, and *Aspergillus* sp. Additionally, we developed a new method for the directional production of cyclic dipeptide backbones. The *criC* gene was successfully expressed in *A. oryzae*, which produced 34 mg/L *Cyclo*-TA when substrates were provided in the culture medium. Microsomal experiments further demonstrated the catalytic effects of CriC in vitro. Overall, we not only elucidated the function of large fragments gene NRPS, but also demonstrated that *A. oryzae* is a powerful tool for the production of complex natural products.

## Material and methods

### General experimental procedures

All reagents commercially supplied were used as received. HPLC analysis was performed using an Agilent 1260 Series with a DAD detector (California, CA, USA). ^1^H- and ^13^C-NMR spectra were recorded on Bruker AVAN CEIII HD 500. Chemical shifts were reported as *δ* scale in ppm as an internal reference (CD_3_OD; ^1^H NMR = 3.31 ppm, ^13^C NMR = 49.0 ppm). Mass spectra were obtained with an AB SCEIX Triple TOF 6600. Column chromatography was carried out on C18 silica gel (Agilent Technologies. USA). Oligonucleotides for polymerase chain reaction (PCR) were purchased from Tsingke Biotechnology Co., Ltd.

*Escherichia coli* DH5α was used for cloning and following standard recombinant DNA techniques. This study used a fungal host strain *A. oryzae* NSAR1, a quadruple auxotrophic mutant (*niaD*-, *sC*-, *∆argB*, *adeA*-) for fungal expression [38]. The transformant was grown on DPY (dextrin-polypeptone-yeast extract: 2 % dextrin, 1 % polypeptone, 0.5 % yeast extract, 100 mL) medium supplemented with appropriate nutrients [39].

### Fungal material, preparation of expression plasmids, and transformation of *A. oryzae*

*Eurotium cristatum* NWAFU-1 was identified by morphological observation and by analysis of the ITS regions of its rDNA (GenBank accession No: OM276864). Genomic DNA of *E. cristatum* NWAFU-1 was prepared according to the literature procedure. The *criC* was amplified with a primer set as shown in Additional file 1: Table S3. PCR reactions were performed with the KOD-Plus-Neo (TOYOBO). PCR product was inserted into the appropriate restriction site KpnI using ClonExpress MultiS One Step Cloning Kit (Vazyme Biotech Laboratories) to construct expression plasmids, pUSA2-*CriC*. The expression vector was sequenced by Sangon BioTech using KBseq technology to obtain the DNA sequence of *criC*, which was submitted to NCBI under the GenBank accession No. OM307404. Transformation of *A. oryzae* NSAR1 was performed by the protoplast-polyethylene glycol method reported previously [39]. pUSA2-*criC* was used for the transformation to construct AO-*criC*.

### Biotransformation and substrate addition

Mycelia of *A. oryzae* transformants were inoculated into 10 mL of MPY (maltose-peptone-yeast extract: 3 % maltose, 1 % polypeptone, 0.5 % yeast extract) medium containing appropriate nutrients in 50 mL Erlenmeyer flasks. After an additional 3 days of incubation at 30 °C, the mycelia were collected by filtration and soaked in acetone (20 mL). The organic layer was then concentrated in vacuo. The crude extracts were analyzed by Agilent 1260 Series HPLC equipped with an Agilent EC-C18 (PorosheII 120, 150 mm × 4.6 mm) at the following conditions: flow rate; 0.6 mL/min, Detection; 280 nm, Solvent system; methanol in H_2_O, 0–16 min, from 5% to 75% linear; 17 min, 100%; 18–22 min 5%.

Substrate addition experimental procedures and subsequent product extraction procedures were the same as described above. Equimolar concentrations of alanine and tryptophan were added to the transformants before induction culture. Quantification of conversion products was achieved by the standard curve method with HPLC. The HPLC analytical method is as described above.

### Microsomal activity assay for AO-*criC* transformant

*A. oryzae* NSAR1 transformant AO*-criC* was grown in 100 mL of MPY medium without methionine at 200 rpm and 30 °C for two days. The mycelium was then collected by centrifugation at 5000*g* for 10 min and ground to a powder in liquid nitrogen. The powder was resuspended in buffer B (0.6 M sorbitol, 0.1 M KCl, 1.0 mM EDTA, 2.0 mM DTT, 1.0 mM PMSF, 50 mM Tris-HCl, pH 7.5) and lysed using an ultrasonic cell disruptor (0 °C for 30 min). The suspension was centrifuged at 8000*g* and 4 °C for 10 min and the supernatant was further separated by ultracentrifugation at 100,000*g* and 4 °C for 1 h. The microsomal precipitate that had settled at the bottom of the centrifuge tube was then resuspended in 1 mL of buffer C (20 % glycerol, 50 mM Tris-HCl, 1.0 mM MgCl_2_, 1.0 mM EDTA, 1.0 mM DTT, pH 7.5) and stored at −80 °C. The mycelium of *A. oryzae* NSAR1 was used to isolate the microsomal fragments and stored at −80 °C.

The catalytic activity test of microsomes containing CriC protein was performed in a total volume of 200 μL reaction mixture, containing 180 μL microsomal fraction of the transformant harboring CriC, 1 mM ATP, and 0.5  mM substrates were incubated at 30 °C for 24 h. As a negative control, the reaction catalyzed by microsomal fraction of *A. oryzae* NSAR1 was also performed with the same reaction mixture and reaction conditions. Subsequently, the reaction mixture was quenched with equal methanol. The reaction mixture is subjected to HPLC detection after 15,000*g* centrifugation for 10 min and filtration (0.22 μM). The reactions were quenched at 4 h, 8 h, 12 h, 16 h, 20 h, and 24 h in time-course experiments.

### Large scale fermentation AO-*criC* transformant

Mycelia of transformant AO-*criC* was inoculated into 100  mL of MPY medium containing appropriate l-Trp and l-Ala in 500 mL Erlenmeyer flasks, and a total of six Erlenmeyer flasks. After incubation at 30 °C for 3 days, the mycelia were collected by filtration and extracted with acetone (400 mL). After filtration, the filtrates were concentrated in vacuo. The residues were resolved in ethyl acetate, and the organic layer was washed with brine and concentrated in vacuo. The crude extracts were purified using silica gel column chromatography (hexane:ethyl acetate, 4:1 to 2:1) to isolate the products.

*Cyclo*-AT: HR-ESI-MS analysis; calcd. for C_14_H_16_N_3_O_2_ [M+H]^+^ calcd: 258.1238, found: 258.1241. [α]_D_^25^ = +17.2 (*c* 0.05, EtOH). The NMR data and spectrums are shown in Additional file 1: Table S2 and Figs. S5–10.

## Supplementary Information

**Additional file 1. **Supplementary experimental section. **Fig. S1.** Mycelial morphology of *E. cristatum* NWAFU-1. **Fig. S2.** Molecular network of the metabolic products from *E. cristatum* NWAFU-1. **Fig. S3.** Proposed biosynthetic gene clusters of echinulin and function analysis of each gene in the BGC. **Fig. S4.** Sequence similarity network analysis of CriC. **Fig. S5.** ^1^H-NMR spectrum of *Cyclo*-TA (CD_3_OD-*d*4, 500 MHz). **Fig. S6.** ^13^C-NMR spectrum of *Cyclo*-TA (CD_3_OD-*d*4, 125 MHz). **Fig. S7.** HSQC spectrum of *Cyclo*-TA (CD**3**OD-*d*4). **Fig. S8.** HMBC spectrum of *Cyclo*-TA (CD_3_OD-*d*4). **Fig. S9.** ^1^H-^1^H COSY spectrum of *Cyclo*-TA (CD_3_OD-*d*4). **Fig. S10.** NOESY spectrum of *Cyclo*-TA (CD3OD-*d4*). **Fig. S11.** HPLC traces of time-course biochemical assays for microsome containing CriC. **Fig. S12.** Substrate promiscuity analysis of CriC. **Fig. S13.** HPLC traces of AO-*criC* product under non-linear increasing concentration gradient substrate feeding. **Fig. S14.** Domain analysis and speculative reaction mechanism for CriC. **Fig. S15.** Genome mining-based CriC uncovered serval BGCs responsible for *Cyclo*-TA containing compounds. **Table S1.** Primers used for construction of expression plasmids. **Table S2.** NMR Data of *Cyclo*-TA in CD_3_OD-*d*4 (500 MHz for ^1^H NMR, 125 MHz for ^13^C NMR). **Table S3.** Percent identity matrix of CriC and its homologies in CriC group branch on the evolutionary tree.

## Data Availability

All data for this study are included in this published article and its additional file.

## Abbreviations

aa-tRNA: Aminoacyl-transfer RNA
BGC: Biosynthesis gene cluster
CDP: Cyclodipeptide synthase
DMA: Dimethylallyl
DKP: Diketopiperazine
FMN: Flavin mononucleotide
GNPS: Global natural products social molecular networking
HPLC: High performance liquid chromatography
HR–ESI–MS: High resolution–electrospray ionization–mass spectrometry
ITS: Internal transcribed spacer
NCBI: National Center for Biotechnology Information
NMR: Nuclear magnetic resonance
NRPS: Nonribosomal peptide synthetase
UPLC–ESI–HRMS: Ultra-high performance liquid chromatography–electrospray ionization–high resolution mass spectrometer
