# Supplementary material for: Efficient production of a cyclic dipeptide (cyclo-TA) using heterologous expression system of filamentous fungus Aspergillus oryzae
Source: Microb Cell Fact. 2022 Jul 18;21:146. doi: 10.1186/s12934-022-01872-8 (PMC9290255; doi:10.1186/s12934-022-01872-8)
Supplement: Supplementary file 1 — Additional file 1. Supplementary experimental section. Fig. S1. Mycelial morphology of E. cristatum NWAFU-1. Fig. S2. Molecular network of the metabolic products from E. cristatum NWAFU-1. Fig. S3. Proposed biosynthetic gene clusters of echinulin and function analysis of each gene in the BGC. Fig. S4. Sequence similarity network analysis of CriC. Fig. S5. 1H-NMR spectrum of Cyclo-TA (CD3OD-d4, 500 MHz). Fig. S6. 13C-NMR spectrum of Cyclo-TA (CD3OD-d4, 125 MHz). Fig. S7. HSQC spectrum of Cyclo-TA (CD3OD-d4). Fig. S8. HMBC spectrum of Cyclo-TA (CD3OD-d4). Fig. S9. 1H-1H COSY spectrum of Cyclo-TA (CD3OD-d4). Fig. S10. NOESY spectrum of Cyclo-TA (CD3OD-d4). Fig. S11. HPLC traces of time-course biochemical assays for microsome containing CriC. Fig. S12. Substrate promiscuity analysis of CriC. Fig. S13. HPLC traces of AO-criC product under non-linear increasing concentration gradient substrate feeding. Fig. S14. Domain analysis and speculative reaction mechanism for CriC. Fig. S15. Genome mining-based CriC uncovered serval BGCs responsible for Cyclo-TA containing compounds. Table S1. Primers used for construction of expression plasmids. Table S2. NMR Data of Cyclo-TA in CD3OD-d4 (500 MHz for 1H NMR, 125 MHz for 13C NMR). Table S3. Percent identity matrix of CriC and its homologies in CriC group branch on the evolutionary tree. [file 12934_2022_1872_MOESM1_ESM.docx]

**Supporting Information**

**Efficient production of cyclic dipeptide (cyclo-TA) using heterologous expression system of filamentous fungus *Aspergillus oryzae***

**Author names and affiliations.**

Jianzhao Qi^1,2^, Haiyan Han^1^, Dan Sui^1^, Shengnan Tan^1^, Changli Liu^1^, Pengchao Wang^1^, Chunliang Xie^3^, Xuekui Xia^4^, Jin-ming Gao^2^*, Chengwei Liu^1^*

1. Key Laboratory for Enzyme and Enzyme-like Material Engineering of Heilongjiang, College of Life Science, Northeast Forestry University, Harbin 150040, Heilongjiang, China.

2. Shaanxi Key Laboratory of Natural Products & Chemical Biology, College of Chemistry & Pharmacy, Northwest A&F University, Yangling 712100, Shaanxi, China.

3. Institute of Bast Fiber Crops, Chinese Academy of Agricultural Sciences, Changsha 410205, Hunan, China

4. Biology Institute, Qilu University of Technology (Shandong Academy of Sciences), Jinan, 250103, Shandong, China.

* Corresponding author: Jin-ming Gao, E-mail: [jinminggao@nwsuaf.edu.cn](mailto:jinminggao@nwsuaf.edu.cn); Chengwei Liu, E-mail: [liuchw@nefu.edu.cn](mailto:liuchw@nefu.edu.cn).

**Table of Contents**

Supplementary experimental section: 3

Supporting Figures:

Figure S1. Mycelial morphology of *E. cristatum* NWAFU-1. 5

Figure S2. Molecular network of the metabolic products from *E. cristatum* NWAFU-1. 6

Figure S3. Proposed biosynthetic gene clusters of Echinulin and function analysis of each gene in the BGC. 7

Figure S4. Sequence similarity network analysis of CriC. 8

Figure S5. ^1^H-NMR spectrum of *Cyclo*-TA (CD_3_OD-*d*4, 500 MHz). 9

Figure S6. ^13^C-NMR spectrum of *Cyclo*-TA (CD_3_OD-*d*4, 125 MHz). 9

Figure S7. HSQC spectrum of *Cyclo*-TA (CD_3_OD-*d*4). 10

Figure S8. HMBC spectrum of *Cyclo*-TA (CD_3_OD-*d*4). 10

Figure S9. ^1^H-^1^H COSY spectrum of Cyclo-TA (CD_3_OD-*d*4). 11

Figure S10. NOESY spectrum of *Cyclo*-TA (CD_3_OD-*d*4). 11

Figure S11. HPLC traces of time-course biochemical assays for microsome containing CriC. 12

Figure S12. Substrate promiscuity analysis of CriC. 13

Figure S13. HPLC traces of AO-*criC* product under non-linear increasing concentration gradient substrate feeding. 14

Figure S14. Domain analysis and speculative reaction mechanism for CriC. 15

Figure S15. Genome mining-based CriC uncovered serval BGCs responsible for *Cyclo*-TA containing compounds. 16

Supporting Tables:

Table S1. NMR Data of *Cyclo*-TA in CD_3_OD-*d*4 (500 MHz for ^1^H NMR ,125 MHz for ^13^C NMR). 17

Table S2. Percent identity matrix of CriC and its homologies in CriC group branch on the evolutionary tree. 18

Table S3. Primers used for construction of expression plasmids. 19

Reference 20

## Supplementary experimental section

**Compound identification based on GNPS analysis**

*Eurotium cristatum* NWAFU-1 was cultured on 80g rice medium in 500 mL Erlenmeyer flasks for 4 weeks at room temperature. Mycelium-covered rice plates were cut into small pieces, and then extracted twice with 200 mL EtOAc, and extracts dried under vacuum. Crude extracts were subjected to UPLC-ESI-HRMS measurements (AB SCEIX Triple TOF 6600) with IDA acquisition modes. MS data preprocessing for feature-based molecular networking was performed using MZmine 2.53. MS data were analyzed via classical or feature-based molecular networking workﬂows, both of which are available in the GNPS web platform (<https://gnps.ucsd.edu>). The network file based on negative-ion mode MS data can be found and accessed at <https://gnps.ucsd.edu/ProteoSAFe/status.jsp?task=69a176c0586549ef9844924fee7e98a4>. Cytoscape V3.8.2 was used for visualization of relationships among compounds with yfileds circle layout.

**Sequence similarity network analysis**

SSN (https://efi.igb.illinois.edu/efi-est/) was used to analysis CriC and its closest homologues in the UniProt database. Filter type was set as E-Value, and filter value was set as 505. Considering that the length of the CriC sequence is 2127 amino acids, the length of the homologous sequence is restricted to 1450-3000 amino acids. Cytoscape V3.8.2 was used for visualization of relationships among CriC and its homologies with yfileds organic layout.

**Phylogenetic analysis of CriC and related enzymes responsible for cyclic dipeptides** **containing Ala or (and) Trp from microorganisms.**

A phylogenetic tree of CriC and proteins associated with tryptophan or alanine involved in the formation of cyclic dipeptides was constructed using the maximum-likelihood method with 3000 bootstrap repeats in MEGA XI. BN1094_02506 (CEE13936.1), Lgor_2215 (KTD01838.1), Lche_2261 (KTC80241.1), Lmac_2531 (KTD24444.1), COCSADRAFT_350779 (EMD58405.1), SI65_10013 (ODM14527.1), ACHE_61021A (BCR91135.1), ASPGLDRAFT_51264 (XP_022397513.1), BO97DRAFT_446073 (XP_025547742.1), PENNAL_c0011G10486 (OQE90703.1), HK57_00375 (KIA75848.1), PENPOL_c014G10511 (OQD61909.1), BDV40DRAFT_314183 (KAE8166787), BDV41DRAFT_590310 (KAE8310535.1), BDW3ODRAFT_246569 (KAB8276265.1), AARAC_006321 (PIG84017.1), BDV24DRAFT_176994 (KAE8346096.1), RCC_09422 (XP_023630432.1), GliP (KAB2575199.1), BKCO1_5300033 (XP_020127225.1), ADK38_06245 (KOG90878.1), DmtB1 (AVP32201), Nvec-CDPS2 (EDO44063), PacA (QEI22736.1), HMPREF1486_06439 (EPD89497.1), NozA ( AKR54045.1), DmtB3 (SFD40868), CDPS_NB5737_ (WP_019889609.1), NascA (AWF71595), CDPS_NB5414_ (WP_063768158.1), HMPREF1486_06439 (EPD89497.1), DtpA (B8NR69), Ao3042_06730 (EIT77215), BvnA (QFZ94974), FtmA (BAH23995), MalG (AGA37267), NotE (E1ACQ0), NotE' (L7WU80), ODM14527 (ODM14527), Pc21g15480 (XP_002568558.1), PhqB (L0E2U2), OkaA (BBB04327), RoqA (B6HJU6), AAY47_15480 (KLU14571.1), Phpb_04530 (OCA52398.1). The entry 545475|MIX10843_1442_75 represents a protein sequence from JGI, the title of this sequence is this entry. The accession NO of the rest of the sequence is the entry for that sequence.

## Supporting Figures:


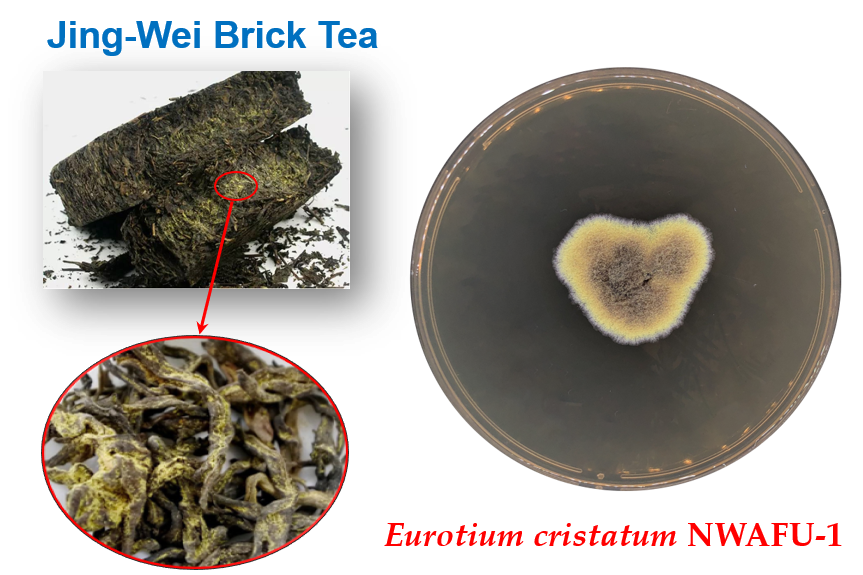


### Figure S1. Mycelial morphology of *E. cristatum* NWAFU-1.

The “golden flower” microbial flora was obtained from the tea cake of Jingwei Fu brick, and the isolate NWAFU-1 was obtained by the method of strain isolation.


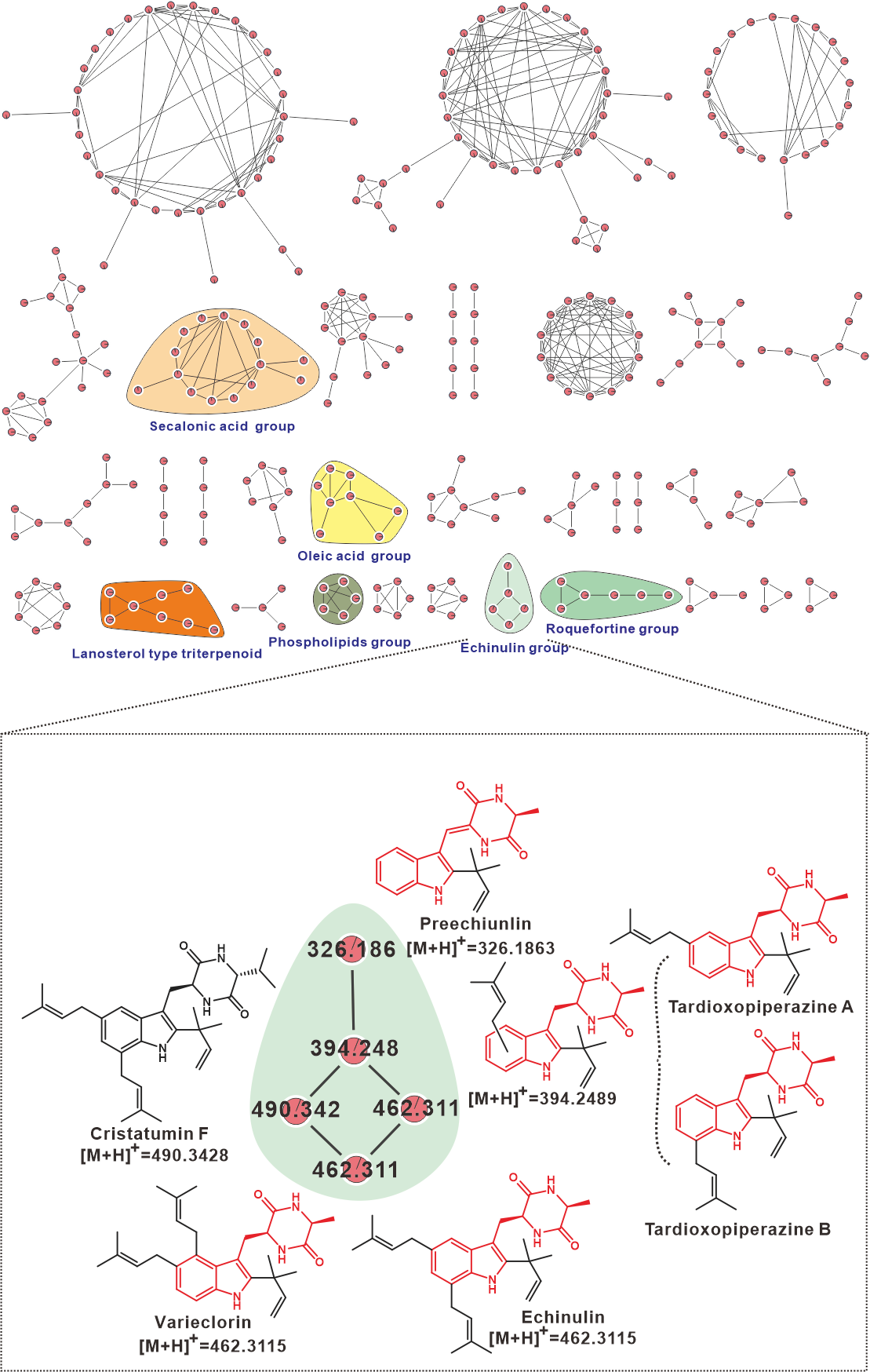


### Figure S2. Molecular network of the metabolic products from *E. cristatum* NWAFU-1.

***Eurotium cristatum***


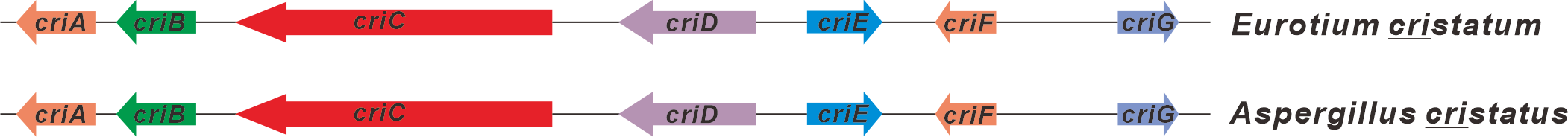


| **Protein** | **Size (AA)** | **Function** | **Relative** **(Identity/ Positives [%])** | **Accession number** |
| --- | --- | --- | --- | --- |
| **CriA** | 431 | Prenyltransferase | Deoxybrevianamide E synthase NotF (51/70) | E0Y3X1.1 |
| **CriB** | 495 | Transporter | High-affinity glucose transporter from Kluyveromyces lactis NRRL Y-1140 (31/49) | P49374.1 |
| **CriC** | 2127 | NRPS | Gliotoxin biosynthesis protein P from *Aspergillus fumigatus* Af293 (32/48) | Q4WMJ7.1 |
| **CriD** | 787 | Unknown | Putative AC transposase from Zeal mays (21/46) | P08770.2 |
| **CriE** | 532 | P450 | Fumitremorgin C synthase (41/59) | B9WZX4.1 |
| **CriF** | 408 | Dimethylallyltryptophan synthase | Tryptophan dimethylallyltransferase CnsF (41/58) | A0A0A2JWD0.1 |
| **CriG** | 421 | FMN oxidoreductase | NADH: flavin oxidoreductase FG08077 (57/70) | I1RV17.1 |

### Figure S3. Proposed biosynthetic gene clusters of Echinulin and function analysis of each gene in the BGC.

Proposed BGC was predicted by 2ndfind (<https://biosyn.nih.go.jp/2ndfind/>) and analyzed by AUGUSTUS.

###
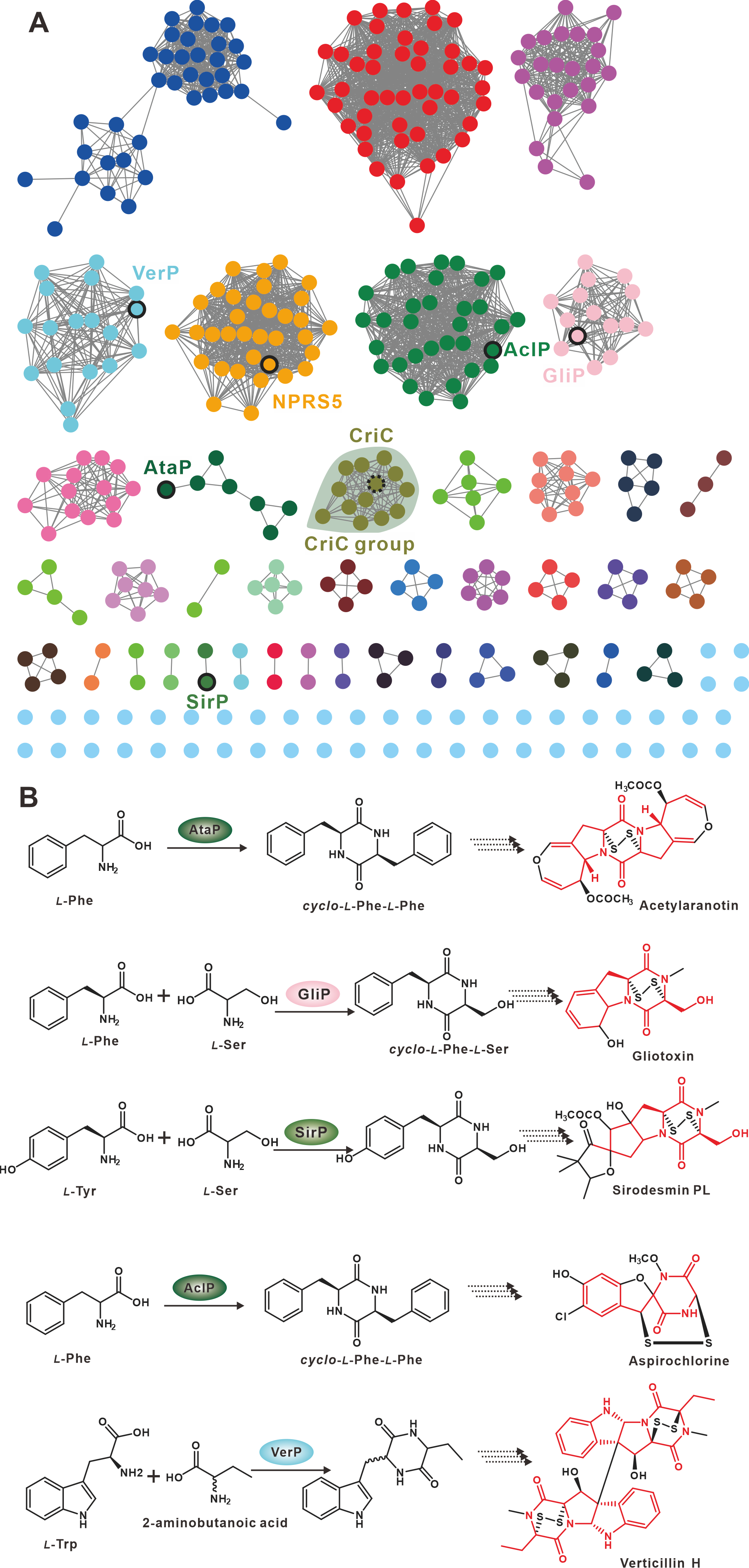
 **Figure S4. Sequence similarity network analysis of CriC.**

**A**: Sequence similarity network analysis based on CriC and its homologous sequences. All homologous sequences were from Uniport Database and the black solid line of dots represents the identified protein sequences. The black dashed dots represent CriC. CriC group was highlighted with a yellow-green background. **B**: Representative NRPS types of cyclic dipeptidases and their catalytic formation of cyclic dipeptides[1-5].

###
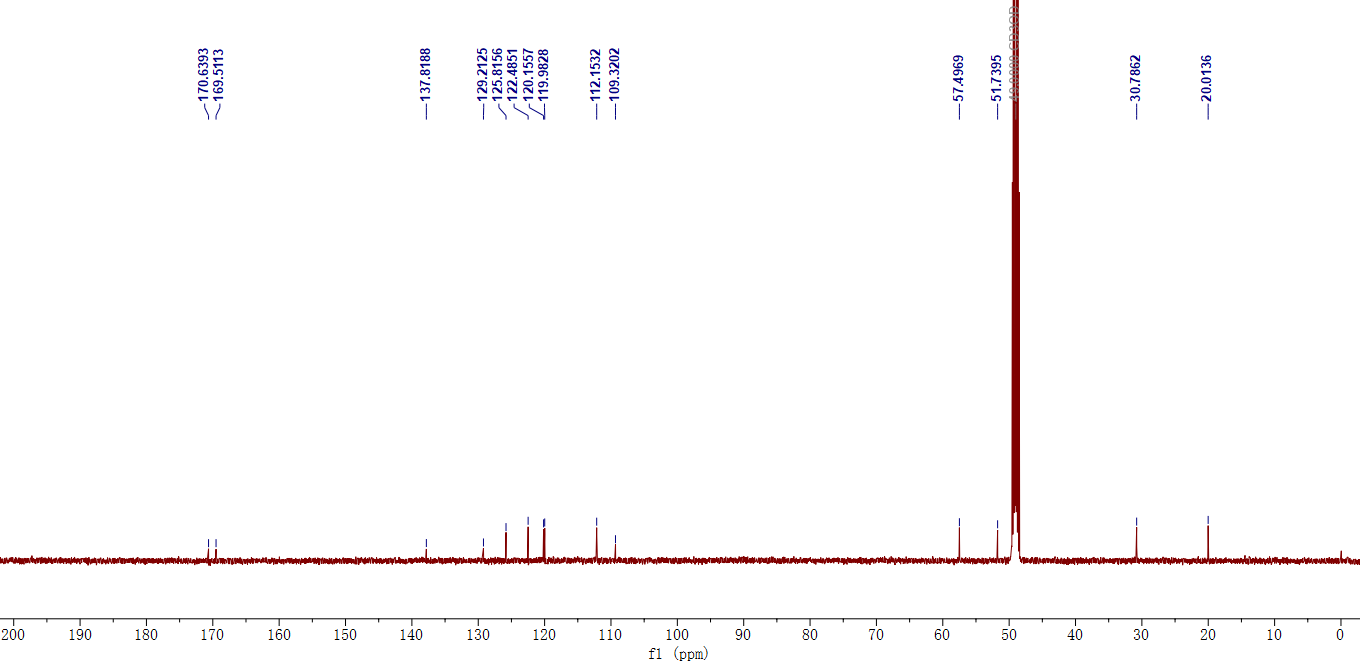
**
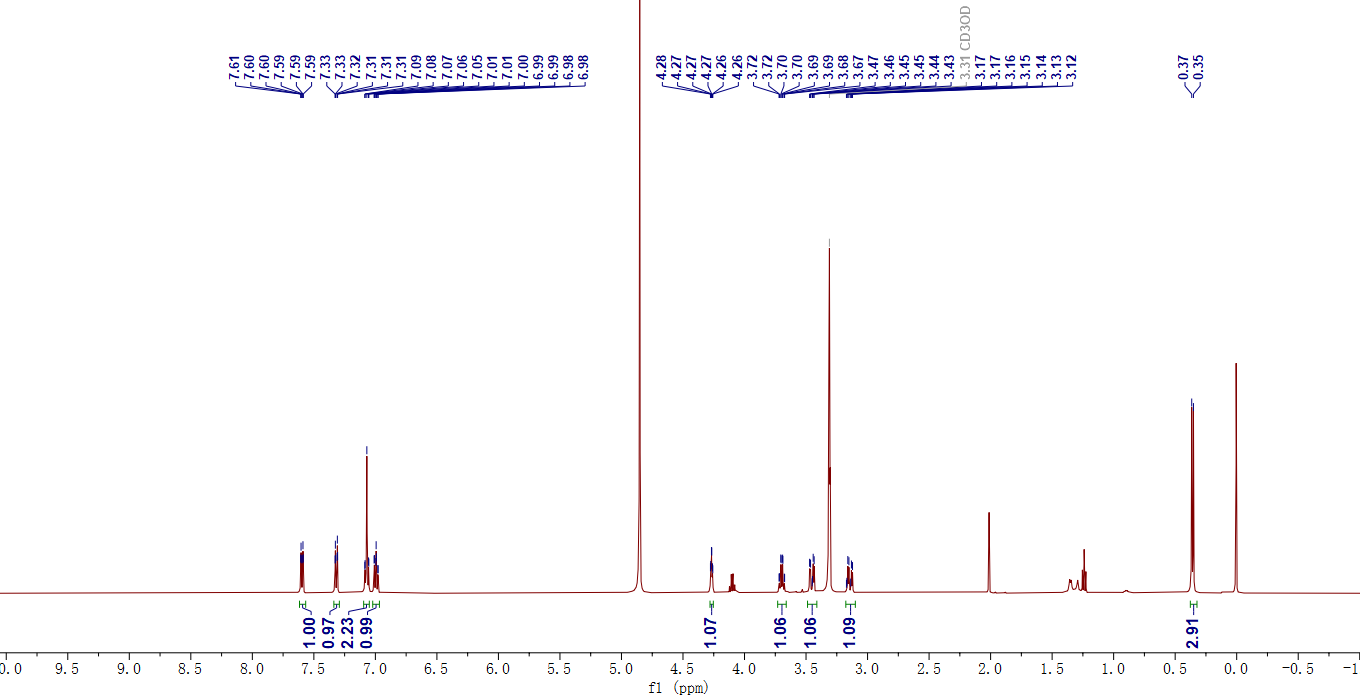
Figure S5.** **^1^H-NMR spectrum of *Cyclo*-TA (CD_3_OD-*d*4, 500 MHz).**

### Figure S6. ^13^C-NMR spectrum of *Cyclo-*TA (CD_3_OD-*d*4, 125 MHz).

###
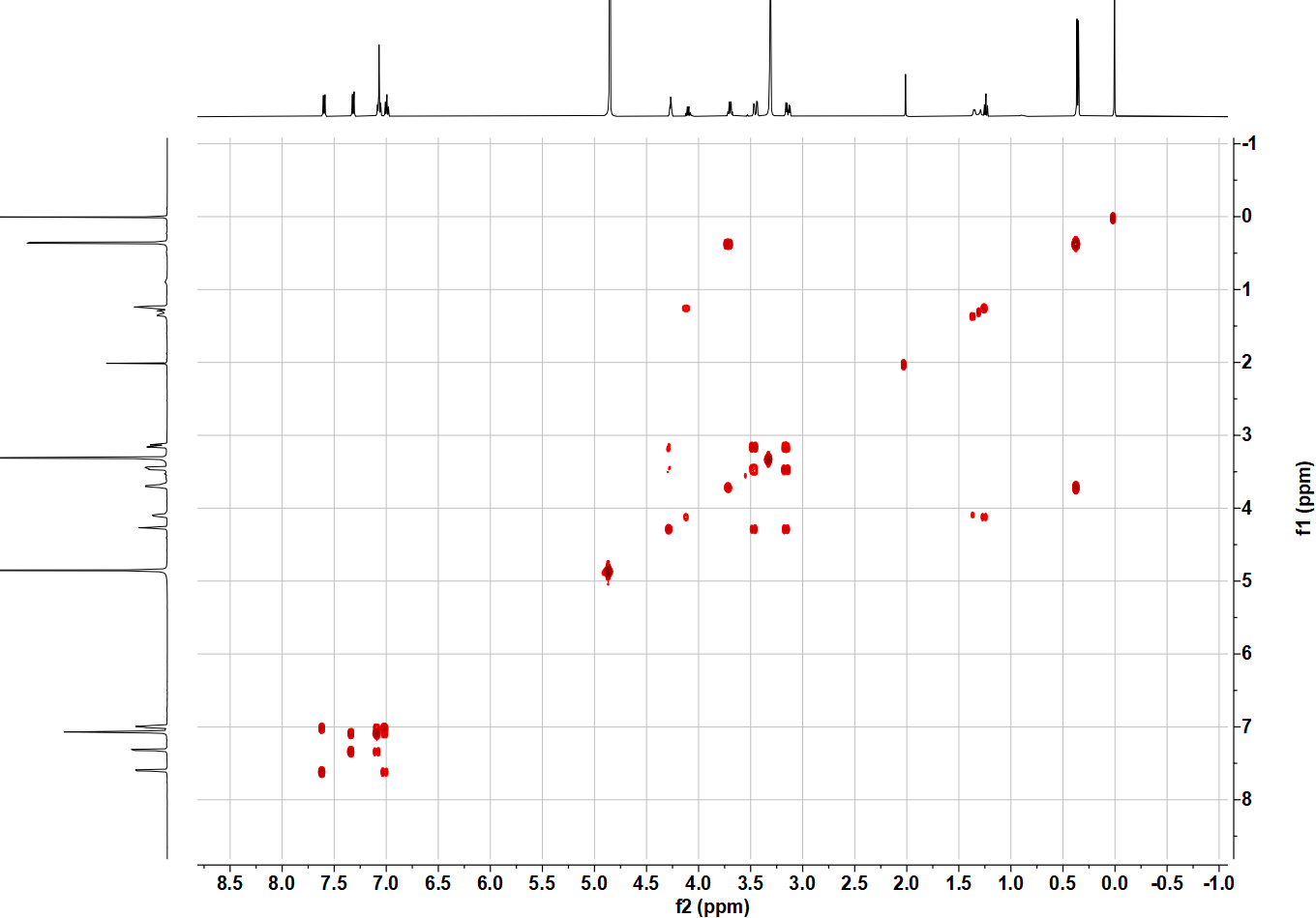

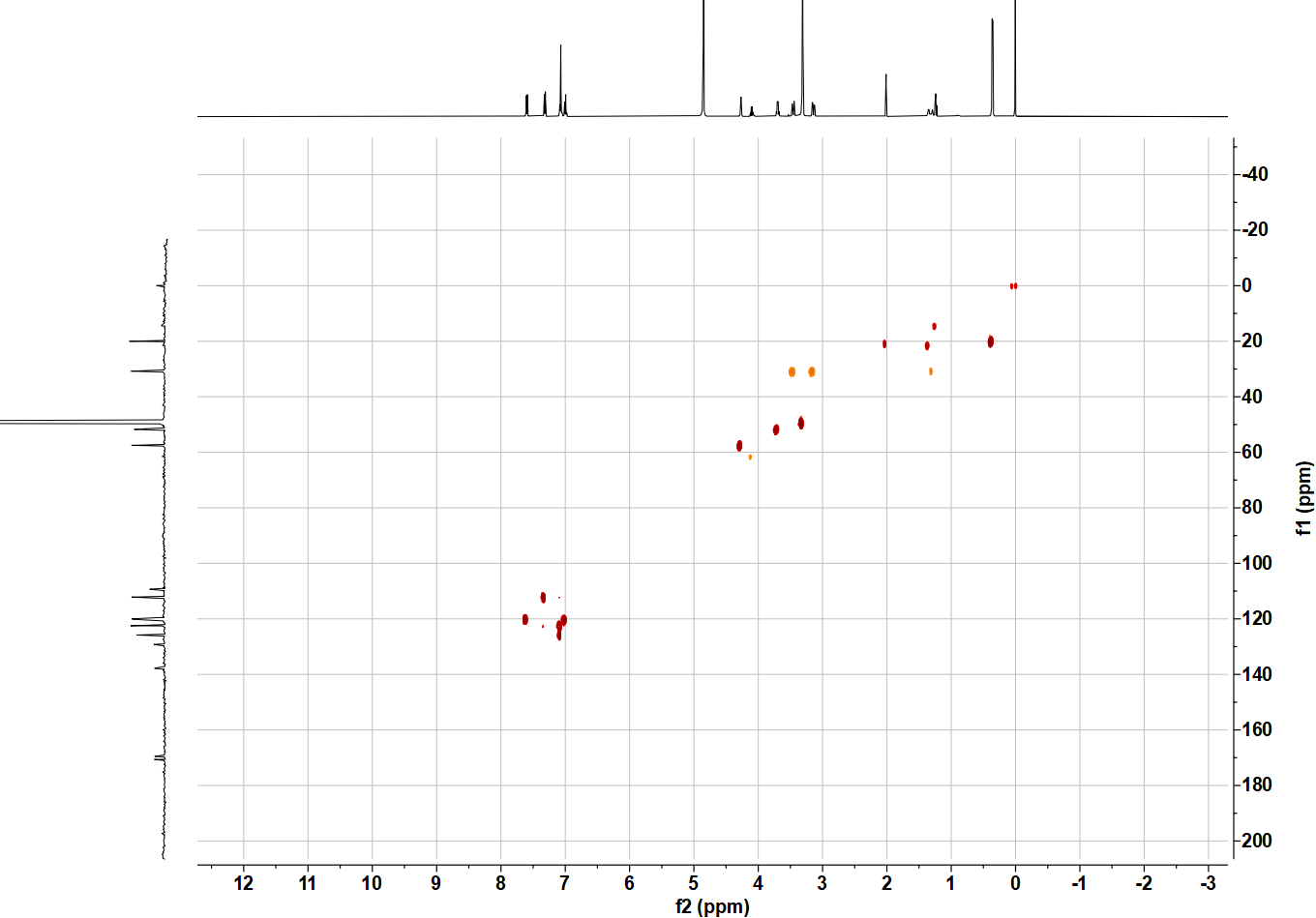
Figure S7. HSQC spectrum of *Cyclo*-TA (CD_3_OD-*d*4).

### Figure S8. HMBC spectrum of *Cyclo*-TA (CD_3_OD-d4).


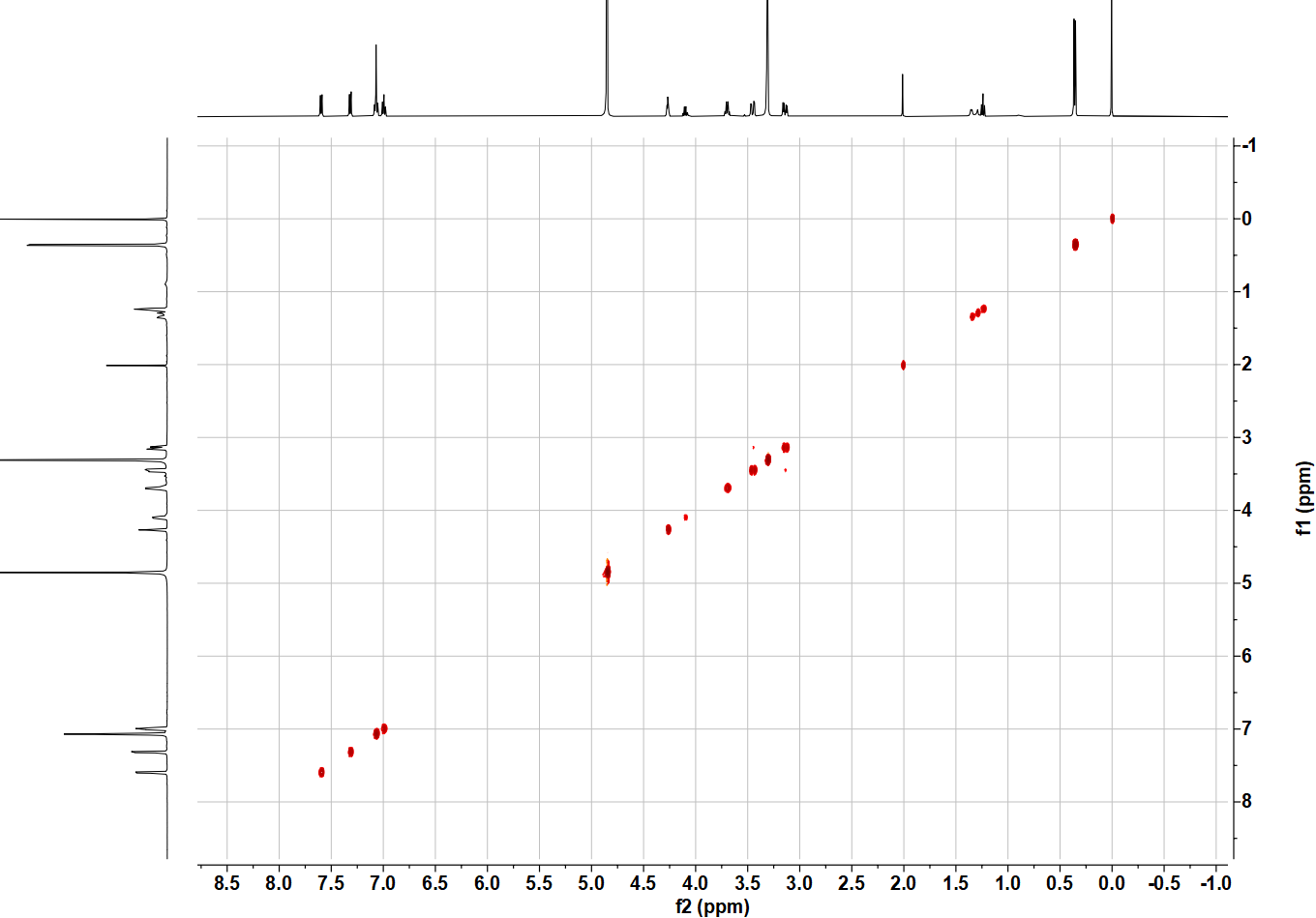
**^
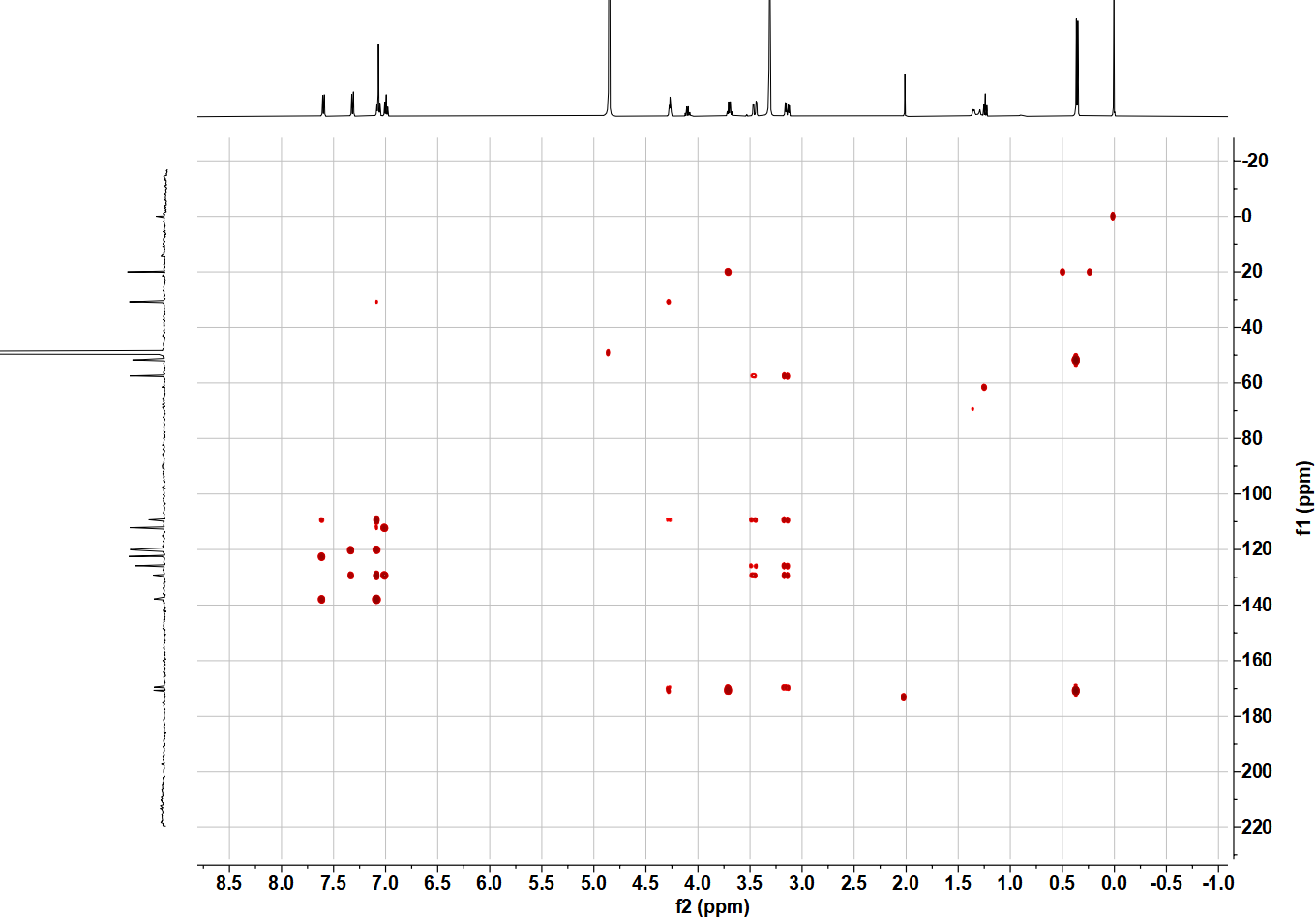
^Figure S9.** **^1^H-^1^H COSY spectrum of *Cyclo*-TA (CD_3_OD-*d*4).**

### Figure S10. NOESY spectrum of *Cyclo*-TA (CD_3_OD-*d*4).

**
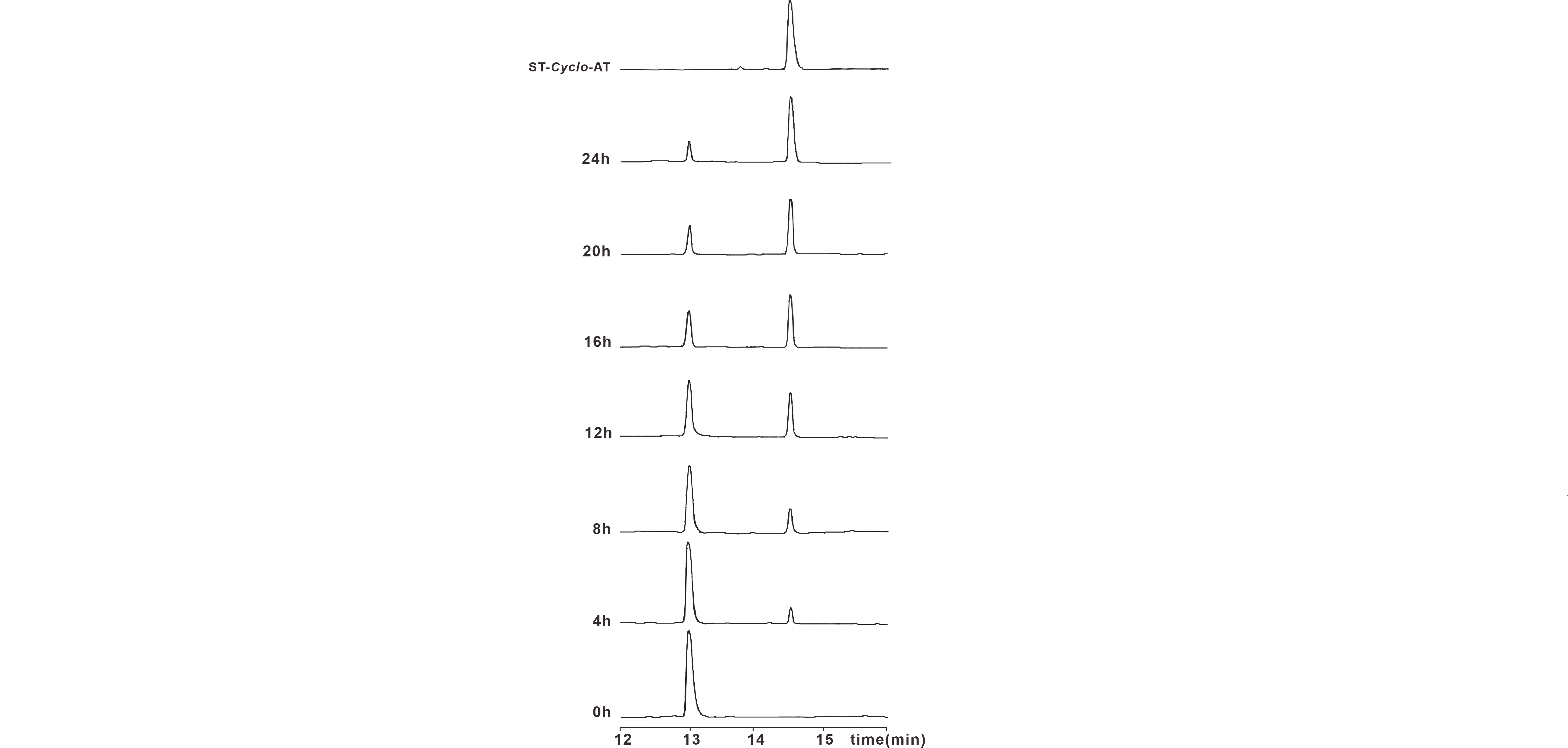
**

### Figure S11. HPLC traces of time-course biochemical assays for microsome containing CriC.

The detection method of HPLC is described in the section "Biotransformation and substrate addition" in the main text.

**
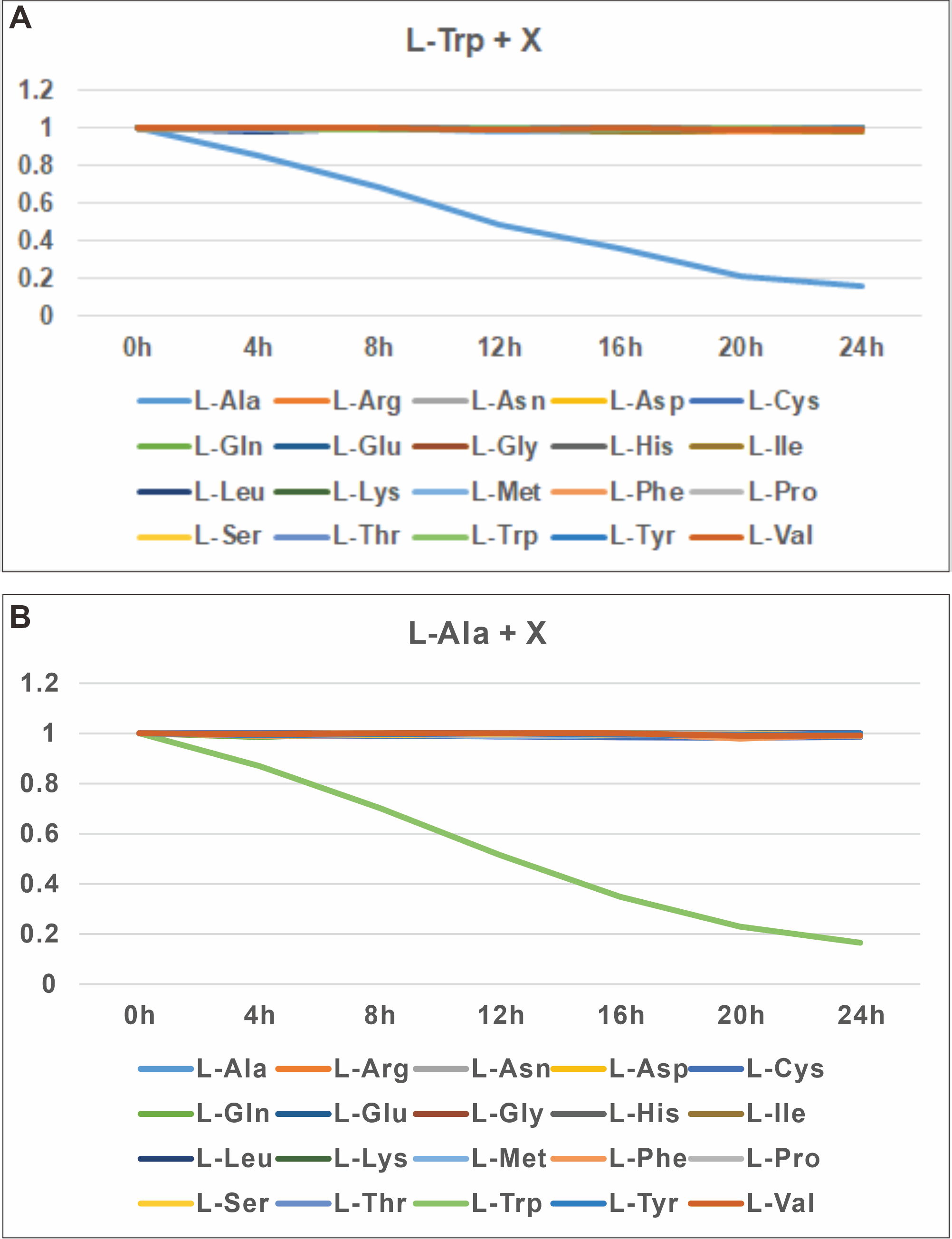
**

### Figure S12. Substrate promiscuity analysis of CriC.

**A**：Trp consumption records for the L-Trp + X reaction system. **B**：Ala consumption records for the L-Ala + X reaction system. Substrate promiscuity analysis of CriC was performed in positive ion mode using an AB SCIEX Triple TOF 6600.


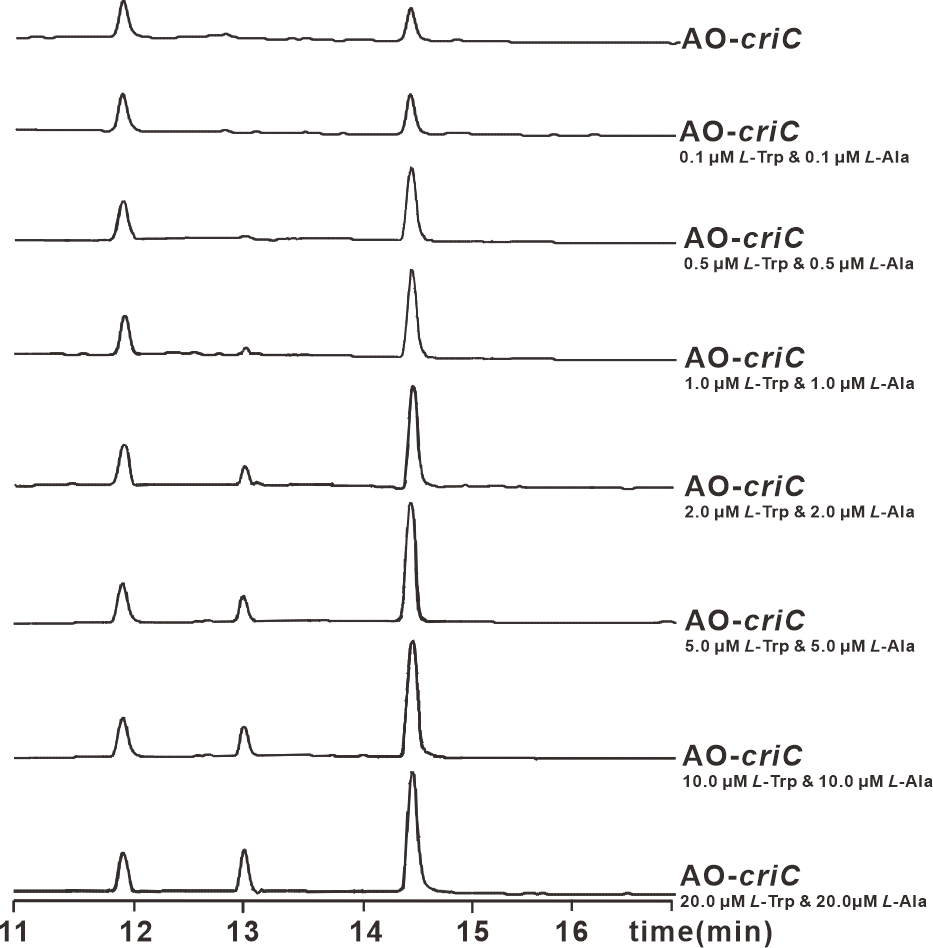


### **Figure S13.** HPLC traces of AO-*criC* product under non-linear increasing concentration gradient substrate feeding.

The detection method of HPLC is described in the section "Biotransformation and substrate addition" in the main text.


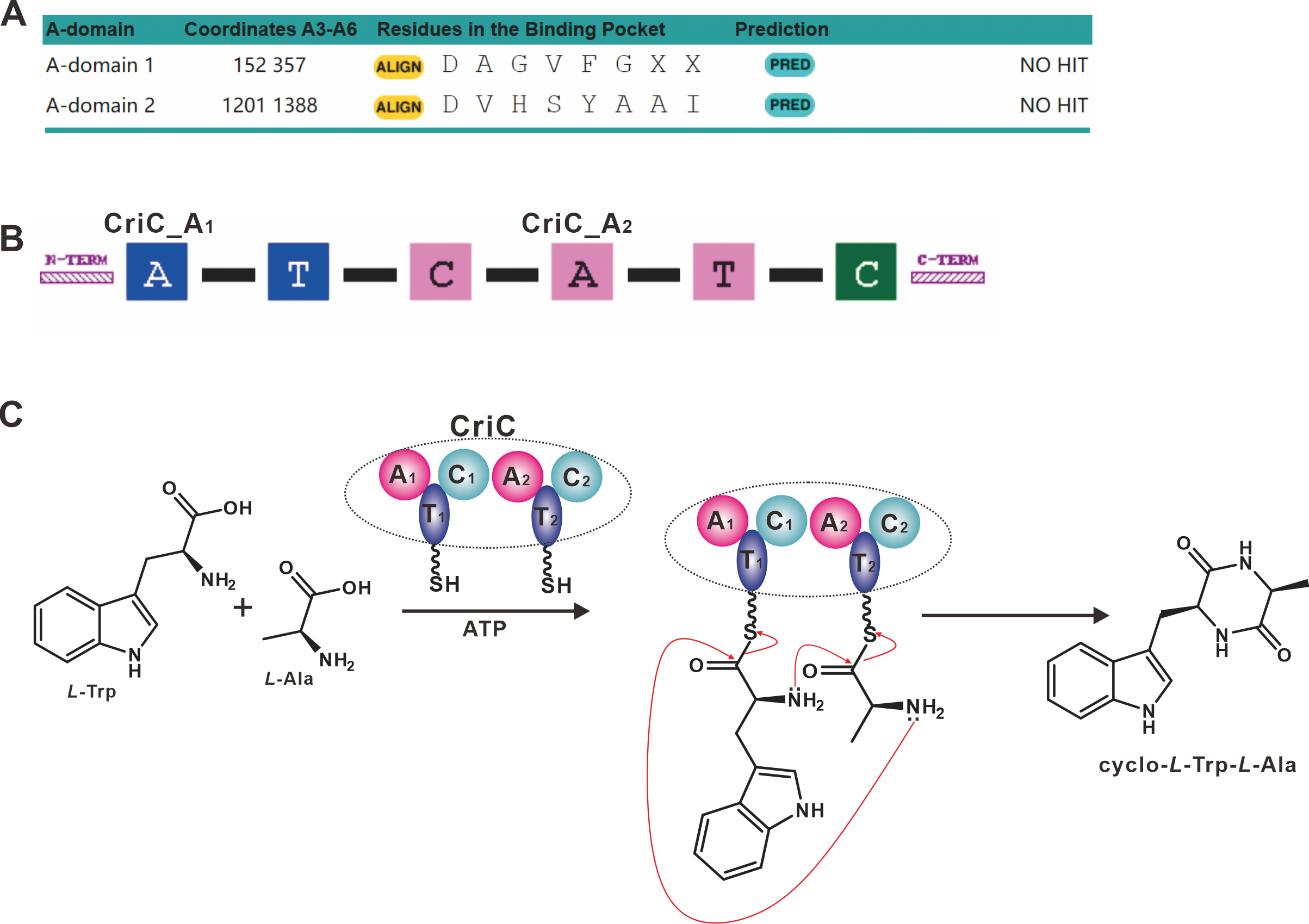


### Figure S14. Domain analysis and speculative reaction mechanism for CriC.

**A**: Adenylation (A) domains in CriC protein were predicted by DOMAIN SEARCH PROGRAM for NRPS (<http://www.nii.ac.in/~zeeshan/search_only_nrps.html>). **B**: CriC functional structural domains were predicted using PKS/NRPS Analysis Web-site (<http://nrps.igs.umaryland.edu>). **C**: Proposed catalytic mechanism of CriC.


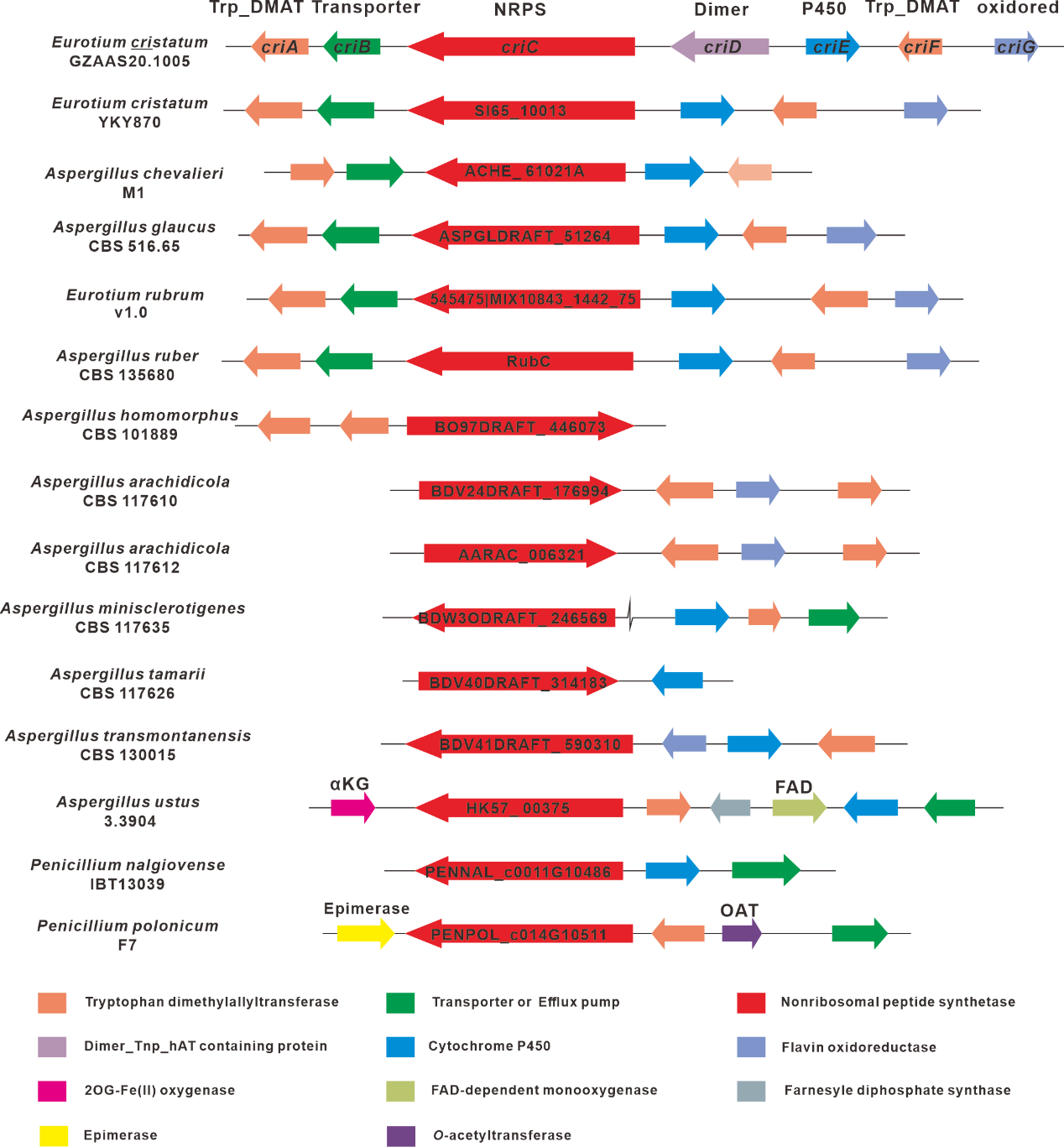


### Figure S 15. Genome mining-based CriC uncovered serval BGCs responsible for *Cyclo*-TA containing compounds.

CriC was used as a probe to identify high similarity homologs of CriC, and then 2ndfind (http://biosyn.nih.go.jp/2ndfind/) was used to predict the region containing high similarity homologs of CriC (5 Kb each before and after) to obtain similar gene clusters.

**Supporting Tables**

### Table S1. NMR Data of *Cyclo*-TA in CD_3_OD-*d*4 (500 MHz for ^1^H NMR ,125 MHz for ^13^C NMR).

| NO. | *δ*_C_ | *δ*_H_ (J in Hz) |
| --- | --- | --- |
| 2 | 125.8 | 7.08, s |
| 3 | 109.3 |  |
| 4 | 119.9 | 7.61, d, *J*=7.9 |
| 5 | 120.0 | 7.01, t, *J*=7.5 |
| 6 | 122.4 | 7.08, t, *J*=7.6 |
| 7 | 112.1 | 7.33, d, *J*=8.1 |
| 8 | 137.8 |  |
| 9 | 129.2 |  |
| 10 | 30.8 | 3.14, dd, *J*=14.6, 4.4  3.46, dd, *J*=14.6, 4.4 |
| 11 | 57.4 | 4.27, t, *J*=4 |
| 13 | 170.6 |  |
| 14 | 51.7 | 3.70, q, *J*=7.0, 14.1 |
| 17 | 20.0 | 0.36, d, *J*=7.0 |
| 16 | 169.5 |  |

### Table S2. Percent identity matrix of CriC and its homologies in CriC group branch on the evolutionary tree.

|  | CriC | SI65-10013 | ACHE-61021A | ASPGLDRAFT-51264 | 545475\|MIX10843-1442-75 | RubC | BO97DRAFT-446073 | BDV41DRAFT-590310 | BDV40DRAFT-314183 | BDW3ODRAFT-246569 | AARAC-006321 | BDV24DRAFT-176994 | PENPOL-c014G10511 | PENNAL-c0011G10486 | HK57-00375 |
| --- | --- | --- | --- | --- | --- | --- | --- | --- | --- | --- | --- | --- | --- | --- | --- |
| CriC | 100.00 | 100.00 | 98.02 | 91.05 | 89.05 | 88.92 | 47.48 | 47.40 | 46.98 | 46.93 | 46.93 | 46.88 | 45.92 | 45.71 | 45.13 |
| SI65_10013 |  | 100.00 | 98.02 | 91.08 | 89.09 | 88.95 | 47.66 | 47.53 | 47.11 | 47.06 | 47.06 | 47.02 | 46.06 | 45.90 | 45.27 |
| ACHE_61021A |  |  | 100.00 | 91.15 | 89.05 | 88.87 | 47.48 | 47.55 | 47.06 | 47.08 | 47.11 | 47.08 | 46.10 | 45.61 | 45.27 |
| ASPGLDRAFT_51264 |  |  |  | 100.00 | 92.59 | 92.48 | 47.14 | 47.61 | 47.12 | 47.19 | 47.27 | 47.39 | 46.41 | 45.98 | 45.24 |
| 545475\|MIX10843_1442_75 |  |  |  |  | 100.00 | 99.86 | 47.20 | 47.77 | 47.30 | 47.40 | 47.46 | 47.40 | 46.63 | 46.27 | 45.00 |
| RubC |  |  |  |  |  | 100.00 | 46.96 | 47.73 | 47.36 | 47.31 | 47.37 | 47.31 | 46.44 | 46.31 | 44.70 |
| BO97DRAFT_446073 |  |  |  |  |  |  | 100.00 | 44.86 | 43.89 | 44.57 | 44.15 | 44.25 | 44.10 | 44.00 | 44.01 |
| BDV41DRAFT_590310 |  |  |  |  |  |  |  | 100.00 | 80.12 | 84.67 | 83.84 | 84.97 | 66.90 | 44.05 | 53.88 |
| BDV40DRAFT_314183 |  |  |  |  |  |  |  |  | 100.00 | 79.66 | 79.04 | 79.72 | 66.02 | 45.06 | 52.31 |
| BDW3ODRAFT_246569 |  |  |  |  |  |  |  |  |  | 100.00 | 90.17 | 90.75 | 67.19 | 44.79 | 53.69 |
| AARAC_006321 |  |  |  |  |  |  |  |  |  |  | 100.00 | 97.31 | 65.37 | 44.68 | 52.77 |
| BDV24DRAFT_176994 |  |  |  |  |  |  |  |  |  |  |  | 100.00 | 66.86 | 44.77 | 52.92 |
| PENPOL_c014G10511 |  |  |  |  |  |  |  |  |  |  |  |  | 100.00 | 42.84 | 52.92 |
| PENNAL_c0011G10486 |  |  |  |  |  |  |  |  |  |  |  |  |  | 100.00 | 43.32 |
| HK57_00375 |  |  |  |  |  |  |  |  |  |  |  |  |  |  | 100.00 |

### Table S3. Primers used for construction of expression plasmids.

| Insert | Restriction 　　　site | Sequence 5’-3’ | Size |
| --- | --- | --- | --- |
|  |  |  | Vector |
| *CriC* | *Kpn*I in lift arm of DNA fragment I | F: aattcgagctcggtaccATGTGAAGCTGATATTCATTC | 3.3 kb |
|  |  | R: gaccaaggtagcgTAGGTAATTGAATGTGACCCTTTTTG | pUSA2 |
|  | *Kpn*I in right arm of DNA fragment II | F: ttacctaCGCTACCTTGGTCCAATATGCA | 3.3 kb |
|  |  | R: tactacagatccccgggtaccCTATACCACCGTTAGATACTGTCGCG | pUSA2 |

## Reference

1. Guo CJ, Yeh HH, Chiang YM, Sanchez JF, Chang SL, Bruno KS, Wang CCC: **Biosynthetic Pathway for the Epipolythiodioxopiperazine Acetylaranotin in *Aspergillus terreus* Revealed by Genome-Based Deletion Analysis.** *Journal of the American Chemical Society* 2013, **135:**7205-7213.

2. Balibar CJ, Walsh CT: **GliP, a multimodular nonribosomal peptide synthetase in *Aspergillus fumigatus*, makes the diketopiperazine scaffold of gliotoxin.** *Biochemistry* 2006, **45:**15029-15038.

3. Stack D, Neville C, Doyle S: **Nonribosomal peptide synthesis in *Aspergillus fumigatus* and other fungi.** *Microbiology-Sgm* 2007, **153:**1297-1306.

4. Wang Y, Hu PJ, Pan YY, Zhu YX, Liu XZ, Che YS, Liu G: **Identification and characterization of the verticillin biosynthetic gene cluster in Clonostachys rogersoniana.** *Fungal Genetics and Biology* 2017, **103:**25-33.

5. Chankhamjon P, Boettger-Schmidt D, Scherlach K, Urbansky B, Lackner G, Kalb D, Dahse HM, Hoffmeister D, Hertweck C: **Biosynthesis of the Halogenated Mycotoxin Aspirochlorine in Koji Mold Involves a Cryptic Amino Acid Conversion.** *Angewandte Chemie-International Edition* 2014, **53:**13409-13413.
